# Human Papillomavirus-Associated Giant Clear Cell Acanthoma and Squamous Cell Carcinoma: A Rare Case Report and Literature Review

**DOI:** 10.3390/jcm13092482

**Published:** 2024-04-24

**Authors:** Roberto Cuomo, Warren M. Rozen, Paola Pentangelo, Alessandra Ceccaroni, Carmine Alfano, Ishith Seth

**Affiliations:** 1 Plastic Surgery Unit, Department of Medicine, Surgery and Neuroscience, University of Siena, 53100 Siena, Italy; 2 Department of Plastic Surgery, Peninsula Health, Melbourne, VIC 3199, Australia; 3 Plastic Surgery Unit, Department of Medicine, Surgery and Dentistry, University of Salerno, 84081 Baronissi, Italy

**Keywords:** clear cell acanthoma, squamous cell carcinoma, inguinal, human papillomavirus, HPV

## Abstract

Clear cell acanthoma (CCA) and squamous cell carcinoma (SCC) represent distinct entities within dermatological oncology, each posing unique diagnostic and therapeutic challenges. CCA is a rare, benign epidermal growth, often not associated with human papillomavirus (HPV) infection, whereas SCC, a more aggressive form of skin cancer, has been linked to both ultraviolet (UV) exposure and HPV. Understanding the co-occurrence of these conditions in a single patient can enhance diagnostic accuracy and therapeutic outcomes. We report a 64-year-old male who underwent an operation for a verruciform lesion in the right groin, which was diagnosed as HPV-positive CCA alongside keratinised SCC. A literature search across January 2024 revealed limited evidence directly linking HPV to CCA, suggesting a need for further investigation. The speculative association between HPV and CCA warrants deeper exploration, especially considering the potential for HPV to contribute to lesion development through indirect mechanisms. The coexistence of CCA and SCC in an elderly patient presents a unique clinical scenario. This emphasises the need for vigilant diagnosis and tailored treatment strategies, highlighting the gap in understanding the pathogenesis of CCA, particularly its potential association with HPV. Further research is crucial for elucidating the complex interactions governing these conditions and for developing targeted interventions.

## 1. Introduction

Clear cell acanthoma (CCA) and squamous cell carcinoma (SCC) pose distinct challenges in dermatological oncology, highlighted by their rarity, varied aetiologies, and aggressive behaviour, particularly in the older male demographic [1]. SCC stands as the second most prevalent skin cancer, primarily driven by excessive ultraviolet (UV) light exposure, with risk factors including immunosuppression, arsenic exposure, and chronic irritation contributing to its development [2,3]. In contrast, CCA is a rare, benign tumour originating from keratinocytes, mainly occurring on the lower limbs but also seen in the inguinal region, trunk, forearms, face, and occasionally the nipple–areola complex [3]. The aetiology of CCA is not well understood, with some proposing it inflammatory dermatosis, evidenced by its psoriasis-like cytokeratin expression [4]. This condition shows a slight male predilection. CCA typically ranges from 0.5 to 2 cm in diameter, though lesions up to 5.5 cm have been reported [5]. The primary treatment is surgical excision, with alternatives like electrocoagulation, cryotherapy, curettage, or carbon dioxide laser excision for multiple lesions or those previously confirmed histologically [5].

The diagnosis of CCA and SCC relies on histopathological analysis, with SCC characterised by keratin pearls and clear cell differentiation, while CCA is identified through its glycogen-rich clear cells and positive periodic acid-Schiff staining [4]. Treatment strategies vary according to the stage of cancer and overall patient health, predominantly involving surgical measures. SCC treatment protocols suggest variable margins based on lesion characteristics, whereas CCA management might include less invasive methods such as cryotherapy, demonstrating the diverse approaches required for these distinct dermatological conditions [6]. 

Moreover, the prognosis for SCC patients remains guarded, with survival rates significantly influenced by the stage at diagnosis, tumour grade, and differentiation level. The rarity of GCCA and its non-regressive nature further complicate the clinical landscape, necessitating ongoing research and case studies to refine understanding and treatment approaches. This juxtaposition of GCCA and SCC within the dermatological oncology field highlights the importance of tailored diagnostic and therapeutic strategies to effectively manage these complex conditions.

## 2. Methodology

### 2.1. Literature Search

A comprehensive literature search was executed across PubMed, Web of Science, Embase and Scopus databases from their inception to January 2024. The search strategy incorporated an array of keywords and terms, including “human papillomavirus”, “HPV”, “clear cell acanthoma”, “squamous cell carcinoma”, “skin cancer”, “HPV-associated skin lesions”, “clear cell acanthoma”, “clear cell acanthosis”, “Degos acanthoma”, and “glycogen-rich acanthoma.” (See Table 1). The intent was to encapsulate a broad spectrum of the literature addressing the nexus between HPV and these specific dermatological conditions, ensuring a comprehensive review that integrates varied nomenclatures and terminologies used in the field. Only articles published in English were considered for inclusion. (Table 2 summarises the most relevant). 

### 2.2. Ethical Considerations 

Written informed consent was obtained from the patient for the use of his clinical information and images for the purposes of research and publication, ensuring respect for his autonomy and privacy. The confidentiality of the patient’s identity and medical information was rigorously protected throughout the study, aligning with ethical guidelines and principles of patient care and research integrity.

### 2.3. Case Presentation

A 64-year-old male patient was referred to the plastic surgery outpatient clinic, presenting with a verruciform lesion in the right groin that had progressively enlarged and darkened over five months. The primary concerns associated with this lesion included pruritus and localised pain. The patient’s medical history was significant for chronic lymphocytic leukaemia and hypertension, which are pertinent given their potential impact on his dermatological health. 

**Figure 1:**
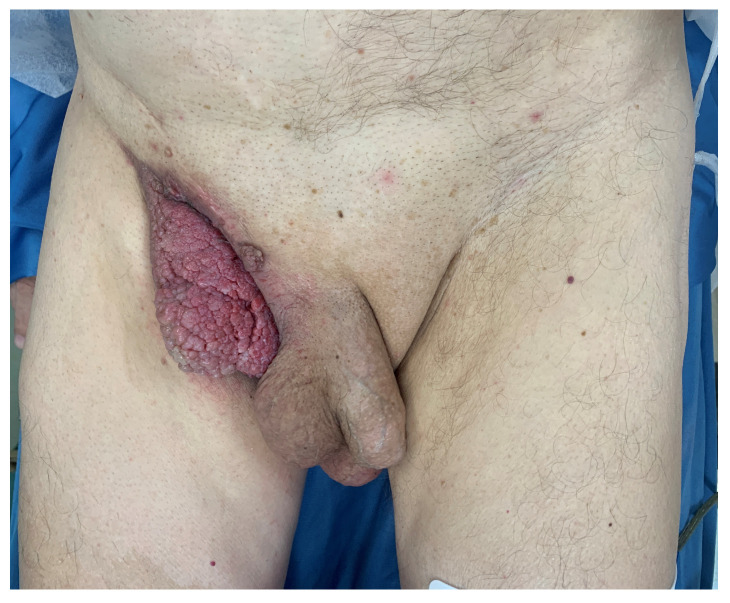
Pre operative picture.

**Figure 2:**
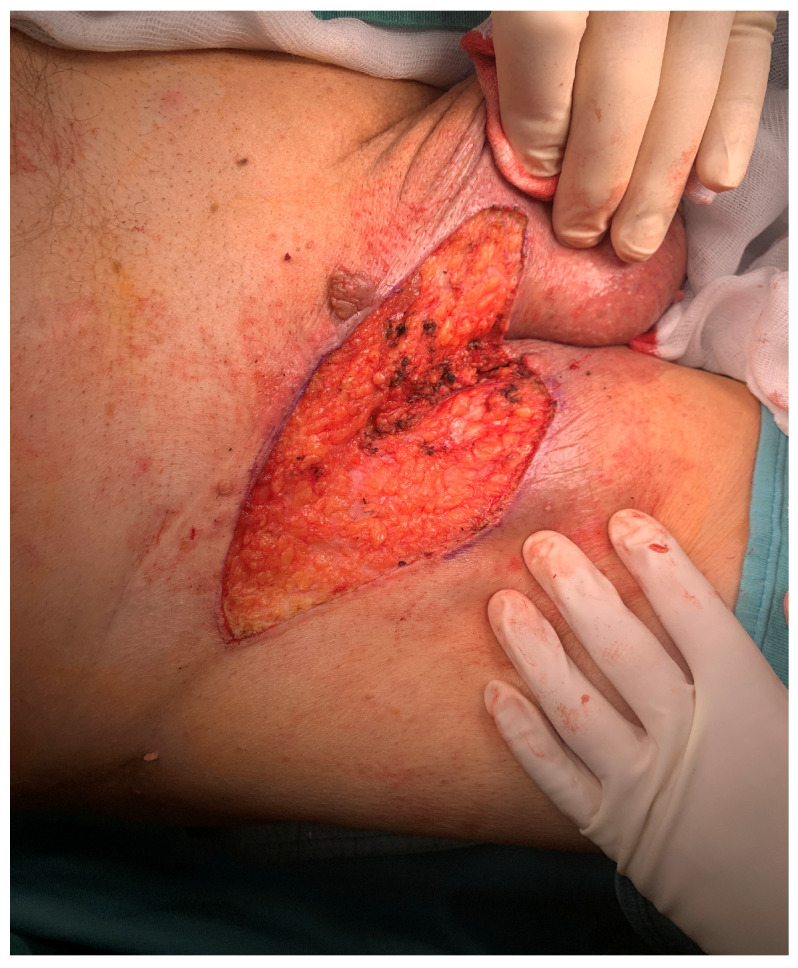
Intra operative picture after acanthoma remotion.

**Figure 3:**
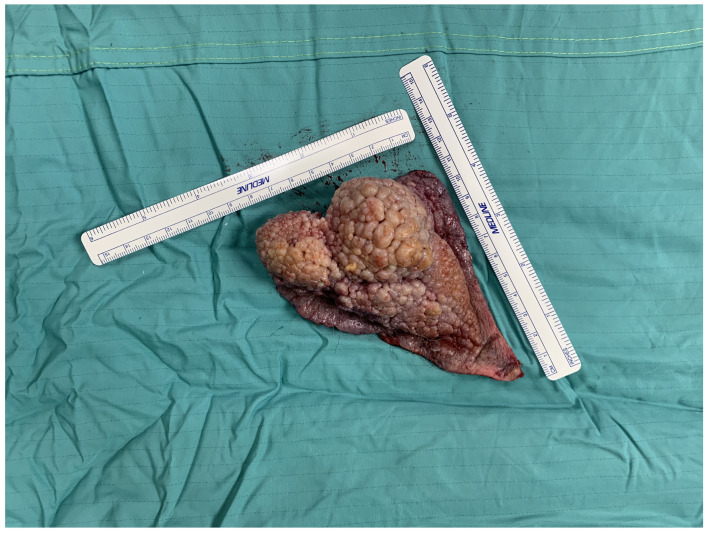
Raised verruciform and mamillated cutaneous neoformation lesion measuring 12 cm in length, 6 cm in width, and 5 cm in height.

Clinically, the lesion was observed as a raised verruciform and mamillated cutaneous neoformation, measuring 12 cm in length, 6 cm in width, and 5 cm in height (Figure 1, Figure 2 and Figure 3). It was characterised by a broad base of implantation and exhibited a taut elastic consistency. Additionally, two minor satellite neoformations were identified at the 3 o’clock and 11 o’clock positions relative to the primary lesion, each measuring 1.5 cm by 1.5 cm and displaying similar morphological features. 

**Figure 4:**
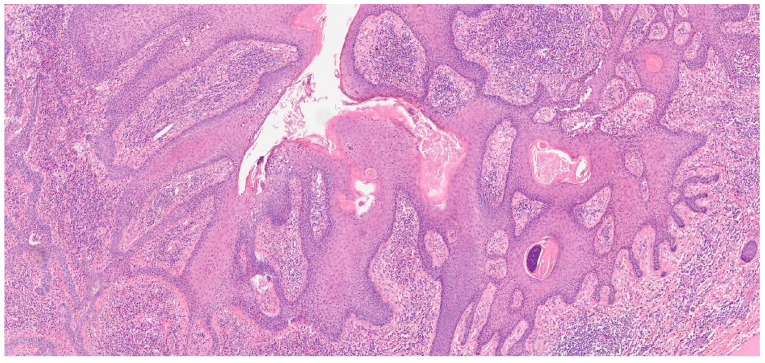
Histopathological evaluation of the lesion with an accumulation of intracytoplasmic glycogen and a predominant presence of eosinophilic granulocytes within intraepidermal micro-abscesses; Immunohistochemical staining demonstrated positivity for p16 indicative of human papillomavirus (HPV) infection.

**Figure 5:**
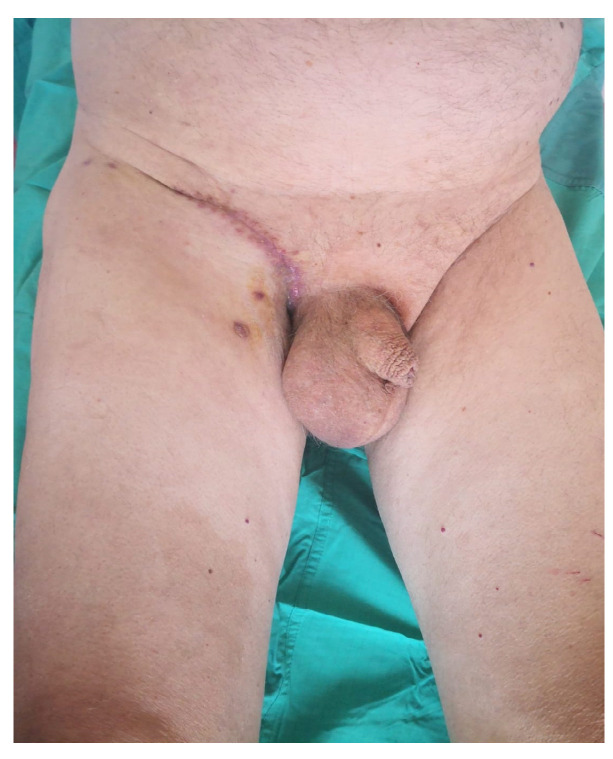
Post-operative picture.

Histopathological evaluation of the lesion revealed significant findings, including an accumulation of intracytoplasmic glycogen and a predominant presence of eosinophilic granulocytes within the intraepidermal micro-abscesses. Further pathological features included dyskeratosis and coilocytotic-like changes. Immunohistochemical staining demonstrated positivity for p16, which is a marker often associated with human papillomavirus (HPV) infection (Figure 4). These findings led to a diagnosis of HPV-positive clear cell acanthoma in conjunction with keratinised squamous cell carcinoma. Additionally, a melanocytic nevus exhibiting severe atypia was identified within the lesion’s composition. The patient underwent surgical treatment involving complete excision of the skin’s neoformation, resulting in a satisfactory functional and cosmetic outcome in the inguinal region at the two-month follow-up and no reoccurrence at the 12-month follow-up (Figure 5). This case highlights the efficacy of surgical intervention in managing extensive dermatological neoformations, emphasising the potential for positive post-operative results both functionally and aesthetically.

## 3. Discussion

This case report and literature review highlight a rare clinical encounter of a 79-year-old male patient presenting with a concurrent diagnosis of inguinal clear CCA devoid of HPV infection and SCC, highlighting the complexity and diagnostic challenges in dermatological oncology. CCA, a benign epithelial tumour of keratinocyte origin, is commonly localised to the lower limbs but can also manifest in regions such as the inguinal area, showcasing its variable clinical presentation. This case is particularly intriguing due to the simultaneous occurrence of CCA and SCC, a common skin cancer known for its aggressive behaviour and association with UV light exposure, in a single patient [29]. This case also underlines the importance of comprehensive histopathological evaluation and the use of ancillary histochemical and immunohistochemical studies in accurately diagnosing and distinguishing between cutaneous malignancies and benign conditions like CCA. The differentiation is crucial for prognostication and selecting an appropriate treatment strategy to ensure optimal patient outcomes. 

CCA’s aetiology remains largely speculative, with the recent literature suggesting it to be a reactive and localised psoriasiform dermatitis rather than a true neoplastic entity. This assertion is supported by its clinicopathologic and immunohistochemical profile, which includes psoriasiform acanthosis, glycogen-rich clear or pale-staining epithelial cells, and a strong immunopositivity for cytokeratin markers akin to those found in psoriasis. Notably, the absence of HPV in the CCA lesion, as indicated by PCR analysis, emphasises the non-viral pathogenesis of CCA, distinguishing it from other HPV-associated dermatoses and neoplasms. 

The association between HPV and clear cell acanthoma CCA remains speculative, with limited direct evidence linking the two. However, a theoretical framework can be proposed based on HPV’s known mechanisms of action in other cutaneous and mucosal pathologies. HPV, particularly its oncogenic strains, possesses the ability to integrate its DNA into the host cell genome, leading to the overexpression of viral oncogenes E6 and E7 [34,35,36]. These oncogenes are known to disrupt the function of tumour suppressor proteins p53 and retinoblastoma, respectively, thereby promoting uncontrolled cell proliferation and potentially leading to neoplastic transformation [35,37,38,39]. In the context of CCA, a benign lesion characterised by glycogen-rich, clear keratinocytes, it is conceivable that HPV could contribute to lesion development through indirect mechanisms [29,40]. For instance, the virus might induce localised inflammatory responses or alter keratinocyte differentiation, creating a microenvironment conducive to the accumulation of glycogen within cells. This process could be facilitated by the disruption of cellular regulatory pathways involved in glycogen metabolism, potentially as a side effect of the host’s attempt to combat viral infection [29,30,41]. Additionally, HPV’s role in modulating immune surveillance could permit the persistence of aberrant keratinocytes, allowing for the manifestation of clear cell pathology observed in CCA. While this hypothesis integrates known aspects of HPV pathogenicity, it is essential to acknowledge the current lack of empirical evidence directly linking HPV to CCA development. Further research, particularly studies exploring the presence of HPV DNA within CCA lesions and investigating the virus’s impact on cellular metabolism and immune response in the skin, is crucial to elucidate any potential causal relationship [42,43,44,45].

The coexistence of CCA and SCC in this patient raises important considerations regarding the differential diagnosis and management of complex skin lesions, especially in elderly patients with significant sun exposure history or other carcinogenic risk factors. SCC, characterised by its potential for aggressive growth and metastasis, necessitates prompt and effective treatment, typically involving surgical excision with adequate margins. In contrast, the treatment approach for CCA, given its benign nature, may involve less invasive options such as cryotherapy or CO2 laser excision, highlighting the need for individualised treatment planning based on the lesion’s nature and the patient’s overall health status [46,47,48]. 

Patients with immunosuppression are at a heightened risk for developing cutaneous malignancies, including SCC, with evidence suggesting roles for both UV radiation and HPV in their pathogenesis [49,50]. Beta-HPVs, in particular, are hypothesised to contribute to cancer development through a ‘hit-and-run’ mechanism, initiating malignant transformation early and becoming less critical as cancer progresses, likely by enabling the accumulation of UV-induced DNA mutations [51]. The World Health Organization has updated its classification for head and neck tumours to include specific sections for HPV-associated dysplasia, acknowledging their distinct clinical attributes from traditional SCCs, yet it stops short of directly associating CCA with HPV infection [52,53,54]. The standardisation of pathology terminology, such as low-grade squamous intraepithelial lesion (LSIL) and high-grade squamous intraepithelial lesion (HSIL), aims to enhance diagnostic accuracy and treatment efficacy, though direct links between HPV and CCA remain unestablished. Epidemiologically, cutaneous HPVs are widespread across healthy skin, possibly constituting part of the normal skin flora, and their role in non-melanoma skin cancer, especially in immunocompromised individuals like organ transplant recipients who are more prone to β-HPV infections, warrants further study [31,55,56,57,58]. A case report on hyaline inclusion acanthoma revealing intracytoplasmic eosinophilic hyaline inclusions in keratinocytes, without association with low- or high-risk HPVs, suggests that alternative oncogenic pathways may be at play [47,59,60]. Advancements in the understanding of the immunopathogenesis of persistent oncogenic HPVs, which may contribute to chronic inflammation and subsequent carcinogenesis, highlight the importance of exploring new diagnostic and therapeutic strategies, including vaccines targeting these viruses. Such advancements offer hope for future management options against the cancers they cause, including skin cancers [61]. 

The relationship between HPV infection and skin cancer, particularly non-melanoma skin cancer (NMSC), such as squamous cell carcinoma (SCC) and keratoacanthoma, has been a subject of considerable scientific inquiry. The systematic review by Neagu et al. (2022) included 2284 patients and found a significant presence of beta and gamma HPV subtypes in various forms of NMSC, suggesting the potential etiological role of HPV in keratinocyte skin cancers [22,62,63,64]. Ramezani et al. in 2020 conducted a meta-analysis specifically examining the association of β-HPV with SCC in immunosuppressed individuals, finding a high prevalence of β-HPVs in cutaneous SCC patients, which supports the hypothesis of β-HPV contributing to SCC development in those with compromised immune systems [65]. Nindl et al. in 2007 and Galloway and Laimins in 2015 discussed the basic virology and clinical manifestations of HPV in relation to NMSC, highlighting the transforming properties of some HPV types and their potential co-factor role in SCC pathogenesis, especially in the context of UV-radiation and immune system interactions [23,24,25,26,27,28,32]. Waterboer et al. in 2008 and Farzan et al. in 2013 provided serological evidence linking beta and gamma HPV types to SCC risk, suggesting a specific association between these HPV genera and skin SCC [24,25]. Conversely, Švajdler et al. in 2016 examined the presence of α-HPV and β-HPV in extragenital/extraungual Bowen’s disease, finding a considerable proportion of lesions positive for high-risk HPV types, which could imply the etiological role of HPV in these precancerous conditions [26]. The literature also discusses the potential benefits of HPV vaccination in preventing NMSC, with some evidence suggesting that a beta-HPV vaccine could serve as an adjuvant treatment measure for patients with recalcitrant NMSC [22,66,67]. The role of HPV proteins in cancer development was explored by Balaji et al. in 2022, who reviewed the mechanisms through which HPV proteins, especially E6 and E7 oncoproteins, contribute to carcinogenesis [68].

The presented data from the literature support the association between HPV, particularly beta and gamma HPV genera, and the development of skin cancer, including SCC and potentially keratoacanthoma. This association is particularly pronounced in immunosuppressed individuals, where the prevalence of β-HPV is significantly higher. The evidence suggests that specific HPV types play a co-factor role in the pathogenesis of skin cancer, potentially through mechanisms involving the suppression of DNA repair and the promotion of oncogenic transformation in the context of UV-induced DNA damage. Despite these associations, the exact causative role of HPV in skin cancer development remains to be fully elucidated, and further research is warranted to understand the complex interplay between HPV infection, immune response, UV exposure, and skin carcinogenesis [33,69]. The potential protective role of HPV vaccination against NMSC also requires additional investigation to determine its efficacy and feasibility as a preventive or adjuvant treatment strategy.

Many authors focused on the relationship between HPV infection and skin cancer, acknowledging its complex and multifaceted nature. HPV is a ubiquitous pathogen with over 200 types, categorised into high risk and low risk based on their oncogenic potential, as described by Kombe Kombe et al. in 2021 [70]. Epidemiological evidence strongly supports the association of specific HPV types, particularly from the beta-genus, with skin cancers such as cSCC in both immunocompetent and immunocompromised individuals, according to Chahoud et al. in 2016 [71,72,73]. The meta-analysis by Chahoud et al. in 2016 provides compelling evidence of a significant association between β-genus HPV and cSCC, with an adjusted pooled odds ratio indicating a moderate but statistically significant risk. Epidermodysplasia verruciformis (EV), a rare genodermatosis caused by mutations in the EVER1 and EVER2 genes, exemplifies the role of HPV in skin carcinogenesis [71,74,75]. Individuals with EV exhibit an abnormal susceptibility to specific HPV types, leading to a high risk of skin carcinoma. This condition highlights the genetic and virological interplay in the pathogenesis of HPV-related skin cancers [7]. Improved detection methods have enhanced our understanding of the prevalence and diversity of cutaneous HPV types in skin tumours, as described by Forslund et al. in 2003 [8]. The presence of multiple HPV types in skin lesions, including those not traditionally associated with high oncogenic risk, suggests the broader involvement of HPV in skin carcinogenesis than previously recognised. Moreover, the concept of “field of cancerisation”, wherein areas of skin exposed to HPV exhibit a predisposition to cancer development, further supports the role of HPV in the aetiology of skin cancers [9,73,76,77]. The presence of EV HPV types in normal skin has been associated with an increased risk of nonmelanoma skin cancer (NMSC), offering a potential predictive value for skin cancer risk [Harwood et al., 2004]. The burden of HPV-associated diseases extends beyond cervical cancer to include a significant impact on skin cancers, particularly in immunosuppressed patients. The high lifetime probability of acquiring HPV underscores the pervasive nature of this infection and its potential to contribute to skin carcinogenesis in a substantial portion of the population [10,11,12,13,14].

This evidence collectively substantiates a significant association between HPV infection and the development of skin cancer, particularly cSCC. The critical roles of specific HPV types, genetic susceptibility (as seen in EV), and immune status in modulating this risk are evident. Given the high prevalence of HPV and the considerable burden of HPV-associated skin cancers, particularly in immunocompromised individuals, these findings underscore the importance of HPV vaccination and targeted prevention efforts for at-risk populations. While the direct causality in some instances remains to be fully elucidated, the aggregate evidence strongly supports the oncogenic potential of HPV in skin carcinogenesis.

The relationship between human papillomavirus (HPV) and acanthoma, particularly epidermolytic acanthoma and its variants, presents a complex and multifaceted area of dermatologic pathology. The role of HPV in the pathogenesis of various skin lesions, including acanthomas, has been a subject of investigation, with some studies identifying HPV DNA in lesions typically associated with epidermolytic changes [78,79]. The term “EV acanthoma” has been coined to describe lesions with histopathological features of epidermodysplasia verruciformis (EV) in the absence of clinical signs of EV, where EV-HPV types have been detected [79,80,81]. This suggests the potential etiological role of HPV in the development of these acanthomas. Notably, a case of HPV-14 and -21-positive EV acanthoma arising in association with condyloma has been reported, highlighting the coexistence of condyloma with EV acanthoma and suggesting a link between HPV infection and the development of these skin lesions. The literature also discusses the utility of in situ hybridisation for detecting genital HPV types in solitary epidermolytic acanthomas, with findings indicating the absence of genital HPV types in these lesions [81,82,83]. This suggests that while HPV may be implicated in the pathogenesis of some acanthomas, its role is not ubiquitous across all types or locations of acanthoma. Moreover, the classification and nomenclature of the oral cavity and mobile tongue tumours have evolved, with the introduction of new entities and the separation of previously grouped conditions reflecting advancements in our understanding of their clinical, histological, and molecular characteristics. This reclassification underscores the importance of precise diagnostic criteria and the need for ongoing research into etiological factors, including the potential role of HPV in these lesions. The evidence presents a dichotomy regarding the association of HPV with acanthomas. While some studies have found HPV DNA in lesions with epidermolytic changes, others have reported negative results for HPV infection in similar cases [84]. This discrepancy may be attributed to differences in lesion types, anatomical locations, HPV detection methods, or the specific HPV subtypes examined.

Many data suggest a correlation between HPV and acanthoma, particularly epidermolytic variants. While HPV DNA has been detected in certain cases, suggesting its possible etiological role, the absence of HPV in other similar lesions indicates that the relationship is not straightforward and may be influenced by multiple factors. Therefore, it is premature to definitively conclude that HPV is a primary causative agent in the development of acanthomas. Further research employing standardised methods for HPV detection across a broader spectrum of acanthoma types and locations is necessary to elucidate the role of HPV in the pathogenesis of these lesions.

The literature on HPV’s role in oncogenesis, particularly in relation to skin cancer, delineates a complex interplay between viral infection and host cellular mechanisms. The oncogenic potential of HPV, primarily high-risk subtypes, has been well established in the pathogenesis of cervical, oropharyngeal, anal, and vulvar cancers [15,16]. This relationship hinges on the ability of HPV to modulate the immune microenvironment towards a protumorigenic state, facilitating immune evasion and suppression. The integration of HPV DNA into the host genome emerges as a pivotal event in tumorigenesis, leading to the enhanced expression of viral oncoproteins and alterations in critical cellular genes [18,85,86]. This may include the disruption of tumour suppressor genes, the amplification of oncogenes, and impairment of DNA repair mechanisms. Notably, recurrent integrations in genes such as RAD51B, NR4A2, and TP63 have been observed, which could result in aberrant protein functions contributing to malignant progression. In the context of skin cancer, specifically squamous cell carcinoma (SCC), the role of HPV is nuanced and less clearly defined. By contrast, HPV infection has been associated with urothelial carcinoma exhibiting squamous differentiation [19,81,84]. New evidence for a direct causative role in skin SCC is less conclusive. However, the expression of p16, a surrogate marker for HPV infection, has been utilised to infer viral involvement in various SCCs, including those of the penis and potentially the skin [20]. This is corroborated by findings that HPV-positive SCCs may exhibit distinct clinical and molecular profiles, including a higher prevalence of non-smokers in HPV-positive cases, suggesting the potential etiologic role of the virus independent of tobacco exposure [19]. The complexity of HPV sequences and integration events, as revealed by whole genome sequencing, further complicates the landscape. In HPV-positive oropharyngeal squamous cell carcinomas, for example, a wide range of HPV copy numbers and disruptive HPV integration events have been observed, leading to significant genomic instability [17,21,87,88,89]. While this study focused on oropharyngeal cancer, the mechanisms of HPV-mediated genomic alteration could plausibly extend to skin SCC, given the commonality in squamous epithelial origin.

This evidence suggests the multifaceted role of HPV in the oncogenesis of various cancers, with specific mechanisms such as viral integration and immune modulation playing pivotal roles. While the direct causative link between HPV infection and skin cancer, particularly squamous cell carcinoma, is less clear and warrants further investigation, the existing literature underscores the potential for HPV to contribute to skin carcinogenesis through similar genomic and immunological pathways observed in other HPV-related malignancies. Targeted therapeutic strategies, including vaccination and immunotherapy, hold promise for the prevention and treatment of HPV-associated cancers, including potential implications for skin cancer management [33,90,91,92,93] (See Supplementary Materials).

## 4. Conclusions

While the current literature provides limited evidence of a direct link between HPV infections and the causation of CCA, there exists a broader connection to skin carcinogenesis in immunocompromised populations, where viruses alongside environmental factors like UV exposure play contributory roles. This underscores the need for further investigation into the intricate interactions that govern these processes and the potential for improved interventions through targeted immunotherapy against oncogenic HPV strains.

Furthermore, the excellent prognosis associated with CCA contrasts with the potentially unfavourable outcome of SCC, depending on its stage at diagnosis. This dichotomy emphasises the necessity for vigilant follow-up and management strategies tailored to the patient’s specific diagnoses, ensuring that the benign nature of CCA does not overshadow the need for the aggressive management of coexistent SCC. In conclusion, the simultaneous occurrence of HPV-clear CCA and SCC in an elderly patient presents a unique clinical scenario that challenges the diagnostic and therapeutic paradigms in dermatology. This case underscores the significance of a thorough clinical and pathological assessment in the management of complex skin lesions, advocating for a multidisciplinary approach to ensure comprehensive care and favourable patient outcomes.

## Figures and Tables

**Table 1:** Literature research strategy.

Search Terms	Databases	Inclusions Criteria	Exclusion Criteria
human papillomavirus	PubMed	English manuscript	Non-English manuscript
clear cell acanthoma	Web of Science		other keywords used
squamous cell carcinoma	Embase		
skin cancer	Scopus		
HPV-associated skin lesions			
clear cell acanthoma			
clear cell acanthosi			
Degos acanthoma			
glycogen-rich acanthoma			

**Table 2:** Analysed manuscripts.

Author	Year	Keypoints
Ramoz [7]	2002	Some mutations can bring verruciformis lesions
Forslund [8]	2003	New strategies to detect HPV
Rohwedder [9]	2008	HPV is linked to acanthoma and other “fields of cancerisation”
Chahoud [2]	2015	HPV genus linked to cutaneous squamous cell carcinoma
Harwood [10]	2004	Increased risk of skin cancer linked to HPV
Kuma [11]	2015	HPV and skin cancer are linked in immunosuppressed patients
Kombe, Kombe [2]	2020	Epidemiology of HPV diseases
Chesson [12]	2014	Prevalence of HPV in the USA
Mahal [13]	2019	Incidence of cancers in HPV patients
Forman [14]	2012	Global burden of HPV and related disease
Roden [2]	2018	HPV vaccination and cancer
Lecher [2]	2022	HPV and oral cancer
Szymonowicz [2]	2020	Analysis of molecular aspects of HPV-related cancer
Moody [2]	2010	HPV oncoproteins
Symer [15]	2018	HPV and anal cancer
Shamseddine [16]	2021	New therapies for HPV-related cancer
Chera [17]	2019	Chemo-radiotherapy for HPV-associated squamous cell carcinoma
Rusan [18]	2015	Genomic aspects of HPV-associated cancer
Kim [19]	2014	P16 immunohistomchemistry expression mlinked to HPV infection in bladder cancer with squamous differentiation
Eich [20]	2020	HPV linked to squamous cell carcinoma of the penis
Gao [21]	2019	Genome sequencing reveals HPV integration in oropharyngea squamous cell carcinomas
Neagu [22]	2023	HPV has a role in skin cancer
Nindl [23]	2007	Description of the role of HPV in non-melanoma skin cancer
Waterboer [24]	2008	Different types of HPV are linked to different types of squamous cell carcinoma of the skin
Farzan [25]	2013	Different types of HPV are linked to different types of squamous cell carcinoma of the skin
Svajdler [26]	2016	HPV has a role in p16 expression in Bowen’s disease
Conforti [27]	2019	HPV can also be linked to keratoacanthoma
Conforti [28]	2019	New concepts of cancerisation of HPV
Parson [29]	1997	SCC can be linked to clear cell acanthoma
D’Antonio [30]	2011	Clinical aspects of rare skin cancers
Bandolin [31]	2020	HPV beta can be linked to tumorigenesis in the skin
Galloway [32]	2015	Describes pathogenesis pathways of HPVs
Balaji [33]	2022	Describes the role of HPV proteins in carcinogenesis
Bouwes [2]	2010	Beta-HPV infection and cutaneous squamous cell carcinoma analogies

## Data Availability

The data that support the findings of the review are available in PubMed, Scopus, EMBASE, and Cochrane Library. No other data are available about the participants of this study.

## Supplementary Materials

The following supporting information can be downloaded at: https://www.mdpi.com/article/10.3390/jcm13092482/s1, Table S1. Literature research strategy. Table S2. Analysed manuscripts—NR = not registered; pts = patients.
