# Supplementary material for: Human Papillomavirus-Associated Giant Clear Cell Acanthoma and Squamous Cell Carcinoma: A Rare Case Report and Literature Review
Source: J Clin Med. 2024 Apr 24;13(9):2482. doi: 10.3390/jcm13092482 (PMC11084788; doi:10.3390/jcm13092482)
Supplement: Supplementary file 1 [file jcm-13-02482-s001.zip › jcm-2959509-supplementary.pdf]

Table S1. Literature research strategy.

Pubmed Search Formula
Search: **((human papillomavirus) OR (HPV,)) OR (clear cell acanthoma)) OR (squamous cell carcinoma)) OR (skin cancer)) OR (HPV-associated skin lesions)) OR (clear cell acanthoma)) OR (lear cell acanthosis)) OR (Degos acanthoma)) OR (glycogen-rich acanthoma)**
Web of Science/EMBASE
((ALL = (human papillomavirus)) OR ALL = (HPV)), AND ALL = (lear cell acanthoma)) AND ALL = (squamous cell carcinoma)) OR ALL = (skin cancer)) OR ALL = (HPV-associated skin lesions)) OR ALL = (clear cell acanthoma)) OR ALL = (clear cell acanthosis)) OR ALL = (Degos acanthoma)) OR ALL = (glycogen-rich acanthoma)
Scopus
TITLE-ABS-KEY ((human OR papillomavirus, OR hpv, OR clear OR cell OR acanthoma, OR squamous OR cell OR carcinoma, OR skin OR cancer, OR hpv-associated OR skin OR lesions, OR clear OR cell OR acanthoma, OR clear OR cell OR acanthosis, OR degos OR acanthoma, OR glycogen-rich OR acanthoma))

Table S2. Analyzed manuscripts—NR = not registered; pts = patients.

Author	Sample Type	Country	Age	Type of Assessment
Parson et al., 1997	Case report	USA	60 years old	Surgical excision
Ramoz et al., 2022	Case series (4 pts)	Colombia, Algeria	NR	Molecular investigation on chromosome 17q25 of verruciform lesions
Forslund et al., 2003	Case series (51 pts)	Australia	NR	PCR to research HPV genoma
Harwood et al., 2004	124 samples by 77 pts	Great Britain	NR	HPV detection in normal skin, benign and malignant conditions
Nindl et al., 2007	Case series 75 pts	Germany	57–58 years old	Viral PCR in human papillomas
Rohwedder et al., 2008	Case series (9 pts)	Germany	44–85 years old	PCR and direct sequencing of HPV in melanomas and non-melanoma skin cancer
Waterboer et al., 2008	120 patients	Germany	NR	Association of acanthoma and carcinoma
Moody et al., 2010	Review (149 manuscripts)	USA	-	Review on pathways of transformation
Bouwes et al., 2010	Multicentric (1534 pts)	Netherlands/Italy	NR	Questionnaire and physical examination
D'Antonio et al., 2011	Case report	Italy	44 years old	Immunohistochemical
Forman et al., 2012	Comprehensive review	France	-	Literature search
Farzan et al., 2013	Population study (1066 patients)	USA	25–74 years old	Interview and serology using venous blood samples
Chesson et al., 2014	Commentary	USA	NR	Author analysis
Kim et al., 2014	Case series (47 pts)	Korea	64–79 years old	Immunohistochemistry expression on squamous cell carcinomas
Kuma et al., 2015	Case series (2 pts)	Japan	43 and 82 yars old	Histopathological examination
Svajdler et al., 2016	Series of 169 specimens	Czech Republic	43–96 years old	PCR on specimens
Symer et al., 2018	Narrative review	USA	NR	Literature analysis

Mahal et al., 2019	Epidemiologic study (12,017 pts)	USA	25–65 years old	Database analysis
Chera et al., 2019	Case series (103 pts)	USA	60 years old (mean)	ctHPV16DNA dPCR assay
Gao et al., 2919	NR	China	NR	RT-PCR and MTT assay on the specimen
Conforti et al., 2019	-	Italy	-	Communication
Kombe, Kombe et al., 2020	Narrative review; 164 manuscripts	China/India	NR	Literature analysis
Szymonowicz et al., 2020	Epidemiological analysis	USA	9–45 years old	Database analysis
Eich et al., 2020	102 pts	USA	50–76 years old	Immunohistochemical analysis on squamous cell carcinomas
Bandolin et al., 2020	Review on 86 manuscripts (1922 pts)	Italy/France	NR	Literature analysis
Shamseddine et al., 2021	NR	USA	NR	Literature analysis
Balaji et al., 2022	Review on 75 manuscripts	India	15–44 years old	Metanalysis of literature data
Neagu et al., 2023	Review (2284 pts)	Italy/Romania	49–78 years old	Literature analysis
